# Supplementary material for: Genome-wide transposon mutagenesis of paramyxoviruses reveals constraints on genomic plasticity
Source: PLoS Pathog. 2020 Oct 9;16(10):e1008877. doi: 10.1371/journal.ppat.1008877 (PMC7577504; doi:10.1371/journal.ppat.1008877)
Supplement: S1 Table — (PDF) [file ppat.1008877.s001.pdf]

S1 Table. Transposon mutagenesis calculations and library metrics of SeV and MuV.

				SeV	MuV
**Calculations**	Genome size including eGFP gene (bp)			16,278	16,314
	Rescue efficiency (events per 10^5 cells) (ref. 28)			4,258	1,007
	Number cells per well			4.0×10^5	
	Number wells transfected			18	42
	Estimated total number of rescue events (ref. 28)			3.1×10^5	1.7×10^5
	Estimated genome coverage (rescue events per genome nt)			x 18.8	x 10.4
**Library Metrics**	***Coverage:** number of nt or codons mutated/ genome size (nt) or total aa number (codons) in genome ORFs	Plasmid Library DNA (6n+18)	nt	**65.9%** (10,141 / 15,384)	**58.9%** (9,060 / 15,384)
			aa	**93.4%** (4,497 / 4,814)	**67.1%** (3,194 / 4,760)
		Rescued virus (P0)	nt	**50%** (7,697 / 15,384)	**20.8%** (3,191 / 15,384)
			aa	**82.3%** (3,963 / 4,814)	**43.3%** (3,194 / 4,760)
	Loss of coverage upon rescue (Plasmid Library minus P0)		nt	**15.9%**	**38.1%**
	**Titer** of rescued virus (iu / mL)			2.2×10^5	3.2×10^2

* Nucleotide coverage is not >90% despite estimated 10X coverage because (a) insertions in the plasmid backbone are not accounted for, which dilutes the calculated coverage in the actual genome, (b) >1 insert per genome results in genomes with variable internal deletions that are not only non-viable, but also cannot be picked up by our sequencing library preparation and analytic pipeline, and (c) Mu-transpogenesis is known not to be completely random. For the abovementioned reasons, we carefully optimized conditions to ensure an estimated 10X coverage if feasible, while minimizing the possibility of having multiple inserts per genome. Despite the lower than expected genome-wide nucleotide coverage in the input library (6n+18), the codon-coverage is high (67.1% to 93.4%).
