# Supplementary material for: Genome-wide transposon mutagenesis of paramyxoviruses reveals constraints on genomic plasticity
Source: PLoS Pathog. 2020 Oct 9;16(10):e1008877. doi: 10.1371/journal.ppat.1008877 (PMC7577504; doi:10.1371/journal.ppat.1008877)
Supplement: S2 Table — (PDF) [file ppat.1008877.s002.pdf]

**S2 Table.** Most highly-represented insertants from SeV library.

| | Nt position in genome[a] | Nt count from region start | Avg number insertants at P2 | Insertant nucleotide sequence[b] | Insertant amino acid sequence[c] |
|---|---|---|---|---|---|
| **N-ORF** | **1405** | **1286** | **15** | **CTAGA\|TGCGGCCGCA\|CTAGA** | **LDAAALE** |
| | **1606** | **1487** | **64** | **TCTGC\|TGCGGCCGCA\|TCTGC** | **SAAAASA** |
| | 1684 | 1565 | 29 | ATAGG\|TGCGGCCGCA\|ATAGG | IGAAAIG |
| **3'UTR-N** | **1694** | **1** | **102** | **TCTAG\|TGCGGCCGCA\|TCTAG** | |
| | **1721** | **28** | **78** | **CTTGA\|TGCGGCCGCA\|CTTGA** | |
| | 1732 | 39 | 457 | TAAGA\|TGCGGCCGCA\|TAAGA | |
| **5'UTR-P** | 1748 | 8 | 127 | GTGAA\|TGCGGCCGCA\|GTGAA | |
| | 1757 | 17 | 266 | CATCC\|TGCGGCCGCA\|CATCC | |
| | **1762** | **22** | **3848** | **ACTGA\|TGCGGCCGCA\|ACTGA** | |
| | 1772 | 32 | 97 | TCAGG\|TGCGGCCGCA\|TCAGG | |
| | 1776 | 36 | 228 | GCAAG\|TGCGGCCGCA\|GCAAG | |
| | 1781 | 41 | 69 | GCCAC\|TGCGGCCGCA\|GCCAC | |
| | 1792 | 52 | 53 | CCCCA\|TGCGGCCGCA\|CCCCA | |
| | 1793 | 53 | 101 | CCCAC\|TGCGGCCGCA\|CCCAC | |
| | **1806** | **66** | **258** | **CCCAG\|TGCGGCCGCA\|CCCAG** | |
| | 1807 | 67 | 61 | CCAGC\|TGCGGCCGCA\|CCAGC | |
| | 1813 | 73 | 87 | GTCGA\|TGCGGCCGCA\|GTCGA | |
| | 1814 | 74 | 130 | TCGAG\|TGCGGCCGCA\|TCGAG | |
| **5'UTR-P** *(C'-ORF)* | 1831 | 91 | 94 | TCGGC\|TGCGGCCGCA\|TCGGC | *SAAAASA* |
| | 1840 | 100 | 425 | ACTTA\|TGCGGCCGCA\|ACTTA | *TLMRPQLT* |
| | 1843 | 103 | 65 | ACCGC\|TGCGGCCGCA\|ACCGC | *TAAAATA* |
| **P-ORF** *(C-ORF)* | **1851** | **8** | **26** | **GATCA\|TGCGGCCGCA\|GATCA** | **DHAAADQ**<br>***WIMRPQIK*** |
| | **2007** | **164** | **22** | **ATCAA\|TGCGGCCGCA\|ATCAA** | **INAAAIN**<br>***PSMRPQST*** |
| **3'UTR-P** | 3601 | 52 | 40 | TCTAC\|TGCGGCCGCA\|TCTAC | |
| **M-ORF** | 4696 | 1028 | 1 | ATCGG\|TGCGGCCGCA\|ATCGG | IGAAAIG |
| **F-ORF** | 6554 | 1689 | 0.7 | CTGAG\|TGCGGCCGCA\|CTGAG | AECGRTE |
| **HN-ORF** | 7168* | 476 | 1.3 | TTCTG\|TGCGGCCGCA\|TTCTG | FCAAAFW |
| **L-ORF** | 12302* | 3747 | 1.7 | CGTGG\|TGCGGCCGCA\|CGTGG | TWCGRTW |

Grey highlighted insertants were rescued and analyzed for growth. Bolded insertants were included in the competition assay.

[a] * indicates that insertant could not be rescued.
[b] Transposon duplicates 5nt from the site of insertion (indicated with vertical bar) and leaves a 10nt scar.
[c] Underlined amino acids were inserted by the transposon. Italicized sequence indicates the alternative C-ORF aa sequence.
