# Supplementary material for: Genome-wide transposon mutagenesis of paramyxoviruses reveals constraints on genomic plasticity
Source: PLoS Pathog. 2020 Oct 9;16(10):e1008877. doi: 10.1371/journal.ppat.1008877 (PMC7577504; doi:10.1371/journal.ppat.1008877)
Supplement: S3 Table — (PDF) [file ppat.1008877.s003.pdf]

S3 Table. Most highly-represented insertants from MuV library.

	Nt position in genome[a]	Nt count from region start	Avg number insertants at P2	Insertant nucleotide sequence[b]	Insertant amino acid sequence[c]
**N-ORF**	1380	1235	1	GGTGG|TGCGGCCGCA|GGTGG	GGAAAGG
	**1386**	**1241**	**2.7**	**CCTGT|TGCGGCCGCA|CCTGT**	**PVAAAPV**
	**1781**	**1636**	**340**	**GGGAG|TGCGGCCGCA|GGGAG**	**VGVRPQGD**
**3'UTR-N**	1840	46	8	CAGGA|TGCGGCCGCA|CAGGA	
	**1858**	**64**	**2**	**GCTAA|TGCGGCCGCA|GCTAA**	
	**1881**	**87**	**21.3**	**TTCCA|TGCGGCCGCA|TTCCA**	
**5'UTR-V/P**	1976	68	7.3	GCAAG|TGCGGCCGCA|GCAAG	
	**1977**	**69**	**1528**	**CAAGC|TGCGGCCGCA|CAAGC**	
	1978	70	5.3	AAGCC|TGCGGCCGCA|AAGCC	
**V-ORF** *(P-ORF)*	1981	3	1	CCATG|TGCGGCCGCA|CCATG	()MCGRTMD
	**1982**	**4**	**1**	**CATGG|TGCGGCCGCA|CATGG**	**()MVRPHMD**
	1984	6	1	TGGAT|TGCGGCCGCA|TGGAT	MDCGRMD
	**2521**	**543**	**1**	**AAGGA|TGCGGCCGCA|AAGGA**	**QGCGRKG** *KDAAAKE*
**F-ORF**	4889*	344	40	GGTGT|TGCGGCCGCA|GGTGT	GVAAAGV
	4904	359	6.7	GCACA|TGCGGCCGCA|GCACA	AHAAAAQ
	4905*	360	1.3	CACAA|TGCGGCCGCA|CACAA	AQCGRTQ
	4910	365	1	GTGAC|TGCGGCCGCA|GTGAC	VTAAAVT
**M-ORF**	4381	1118	2	ATGAG|TGCGGCCGCA|ATGAG	MSAAAMR
**HN-ORF**	7084*	471	25	TTTCT|TGCGGCCGCA|TTTCT	DFLRPHFS
**L-ORF**	12587*	4150	10	ACCTG|TGCGGCCGCA|ACCTG	RPVRPQPV

Grey highlighted insertants were rescued and analyzed for growth. Bolded insertants were included in the competition assay.

[a] * indicates that insertant could not be rescued.

[b] Transposon duplicates 5nt from the site of insertion (indicated with vertical bar) and leaves a 10nt scar.

[c] Underlined amino acids were inserted by the transposon. Italicized sequence indicates the edited P-ORF aa sequence. () indicate that edited sequence contains non-coding sequence.
