# Supplementary material for: Genome-wide transposon mutagenesis of paramyxoviruses reveals constraints on genomic plasticity
Source: PLoS Pathog. 2020 Oct 9;16(10):e1008877. doi: 10.1371/journal.ppat.1008877 (PMC7577504; doi:10.1371/journal.ppat.1008877)
Supplement: S4 Table — (PDF) [file ppat.1008877.s004.pdf]

S4 Table. Transposon mutagenesis calculations and library metrics in NDV.

<table>
<tr><th></th><th colspan="3"></th><th>NDV</th></tr>
<tr><td rowspan="6">Calculations</td><td colspan="3">Genome size
including eGFP gene (bp)</td><td>15,942</td></tr>
<tr><td colspan="3">Rescue efficiency
(events per 10^5 cells) (ref 28)</td><td>375</td></tr>
<tr><td colspan="3">Number cells per well</td><td>4.0×10^5</td></tr>
<tr><td colspan="3">Number wells transfected</td><td>54</td></tr>
<tr><td colspan="3">Estimated total number
of rescue events (ref. 28)</td><td>8.1×10^4</td></tr>
<tr><td colspan="3">Estimated genome coverage
(rescue events per genome nt)</td><td>x 5.1</td></tr>
<tr><td rowspan="6">Library Metrics</td><td rowspan="4">Coverage:
number of nt or codons mutated/ genome size (nt) or total aa number (codons) in genome ORFs</td><td rowspan="2">Plasmid DNA
(6n+18)</td><td>nt</td><td>51.5%
(7,792 / 15,130)</td></tr>
<tr><td>aa</td><td>78.6%
(3,606 / 4,588)</td></tr>
<tr><td rowspan="2">Rescued virus
(P0)</td><td>nt</td><td>34.4%
(5,205 / 15,130)</td></tr>
<tr><td>aa</td><td>61.5%
(2,820 / 4,588)</td></tr>
<tr><td colspan="2">Loss of coverage upon rescue
(Plasmid Library minus P0)</td><td>nt</td><td>17.1%</td></tr>
<tr><td colspan="3">Titer of rescued virus (iu / mL)</td><td>1.0×10^1</td></tr>
</table>
