# Supplementary material for: Genome-wide transposon mutagenesis of paramyxoviruses reveals constraints on genomic plasticity
Source: PLoS Pathog. 2020 Oct 9;16(10):e1008877. doi: 10.1371/journal.ppat.1008877 (PMC7577504; doi:10.1371/journal.ppat.1008877)
Supplement: S5 Table — (PDF) [file ppat.1008877.s005.pdf]

S5 Table. Most highly-represented insertants from NDV library.

	Nt position in genome[a]	Nt count from region start	Average number of insertants at P2	Insertant nucleotide sequence[b]	Insertant amino acid sequence[c]
F-ORF	**5367**	**880**	**49**	**CGGGA|TGCGGCCGCA|CGGGA**	**GMRPHGNL**
	5383	896	442	ATGCG|TGCGGCCGCA|ATGCG	MRAAMRA
	**5384**	**897**	**40**	**TGCGT|TGCGGCCGCA|TGCGT**	**RCGRMRA**
L-ORF	11643	3319	93	CTTAG|TGCGGCCGCA|CTTAG	LVRPHLVS
	11867	3543	48	ATGAC|TGCGGCCGCA|ATGAC	DCGRNDT
	11868	3544	122	TGACA|TGCGGCCGCA|TGACA	DMRPHDTS
	11869	3545	281	GACAC|TGCGGCCGCA|GACAC	DTAAADTS
	11870*	3546	8857	ACACC|TGCGGCCGCA|ACACC	TCGRNTS
	11871	3547	830	CACCA|TGCGGCCGCA|CACCA	TMRPMTSK
	11872*	3548	65000	ACCAG|TGCGGCCGCA|ACCAG	TSAAATSK
	11872[#] + F^{rev}	***3548***	***65000***	***ACCAG|TGCGGCCGCA|ACCAG***	***TSAAATSK***
	11873	3549	868	CCAGC|TGCGGCCGCA|CCAGC	SCGRTSK
	11874*	3550	810	CAGCA|TGCGGCCGCA|CAGCA	SMRPHSKN
	11875	3551	727	AGCAA|TGCGGCCGCA|AGCAA	SNAAASKN
	11876	3552	269	GCAAG|TGCGGCCGCA|GCAAG	KCGRSKN
	11877	3553	84	CAAGA|TGCGGCCGCA|CAAGA	KMRPHKN
	12416	4092	49	TTGGG|TGCGGCCGCA|TTGGG	GCGRIGV
	12727	4403	44	GGAGC|TGCGGCCGCA|GGAGC	AAAAAAAL
	13213	4889	192	CCATT|TGCGGCCGCA|CCATT	PFAAAPLL
	13215*	4891	3286	ATTAC|TGCGGCCGCA|ATTAC	LLRPQLL
	14818	6494	34	ATTAC|TGCGGCCGCA|ATTAC	ITAAAIT

Grey highlighted insertants were rescued and analyzed for growth.
Bold insertants demonstrated syncytia and continuous GFP spread after rescue.
[a] * indicates that insertant did not demonstrate syncytia or GFP spread after rescue.
[#] indicates the insertant rescued with the fusion-revertant compensatory point mutations
[b] Transposon duplicates 5nt from the site of insertion (indicated with vertical bar) and leaves a 10nt scar.
[c] Underlined amino acids were inserted by the transposon.
