# Supplementary material for: Genome-wide transposon mutagenesis of paramyxoviruses reveals constraints on genomic plasticity
Source: PLoS Pathog. 2020 Oct 9;16(10):e1008877. doi: 10.1371/journal.ppat.1008877 (PMC7577504; doi:10.1371/journal.ppat.1008877)
Supplement: S6 Table — (PDF) [file ppat.1008877.s006.pdf]

S6 Table. Primers for generation of insertant clones.

Virus	Insertant	Primer sequence (5' to 3')
SeV	1405-N	For: TGCGGCCGCACTAGAAACAGAAGCTCATGCGGAC
		Rev: TCTAGTGCGGCCGCATCTAGGTCGATATCGGCATTG
	1606-N	For: TGCGGCCGCATCTGCAACCCATGGAGATGAAGGCCGC
		Rev: GCAGATGCGGCCGCAGCAGAATCCTCTTGCCGT
	1684-N	For: TGCGGCCGCAATAGGAGGAATCTAGGATCATACGAGG
		Rev: CCTATTGCGGCCGCACCTATCCCAGCTACTGCTG
	1694-UTR-N	For: TGCGGCCGCATCTAGGATCATACGAGGCTTCAAGGTAC
		Rev: CTAGATGCGGCCGCACTAGATTCCTCCTATCCCAGCT
	1721-UTR-N	For: TGCGGCCGCACTTGATCCGTAGTAAGAAAAACTTAGGGT
		Rev: TCAAGTGCGGCCGCATCAAGTACCTTGAAGCCTCGTA
	1762-UTR-N	For: TGCGGCCGCAACTGATCGGCTCAGGCAAGGCCACA
		Rev: TCAGTTGCGGCCGCATCAGTGGATGAACTTTCACCC
	1772-UTR-P	For: TGCGGCCGCATCAGGCAAGGCCACACCCAAC
		Rev: CCTGATGCGGCCGCACCTGAGCCGATCAGTG
	1806-UTR-P	For: TGCGGCCGCACCCAGCAGTCGAGACAGCCAC
		Rev: CTGGGTGCGGCCGCACTGGGTGTGGTCGGTG
	1840-UTR-P	For: TGCGGCCGCACTTACCGCATGGATCAAGATGCCTT
		Rev: GTAAGTGCGGCCGCAGTAAGTGTAGCCGAAGCC
	1851-P	For: TGCGGCCGCAGATCAAGATGCCTTCATTCTTAAAGAAGATTCT
		Rev: TGATCTGCGGCCGCATGATCCATGCGGTAAGTGTAG
	2007-P	For: TGCGGCCGCAATCAACACTCCCCAAGGACCA
		Rev: TTGATTGCGGCCGCATTGATGGTGTTGTGGAGCC
	4696-M	For: TGCGGCCGCAATCGGAAGGATCAGAAAGCTGTAAAT
		Rev: CCGATTGCGGCCGCACCGATGTTCTTAGCCACAACA
	6554-F	For: TGCGGCCGCACTGAGAAAAGATGATCACGACCATTAT
		Rev: CTCAGTGCGGCCGCACTCAGCCATCGCATCAAACC
	7168-HN	For: TGCGGCCGCATTCTGGAGATGCCCTGTCGGAGAAC
		Rev: CAGAATGCGGCCGCACAGAAACTATGTGGCTCAAGTG
	12302-L	For: TGCGGCCGCACGTGGGCCTACGGGACTGATGAGAT
		Rev: CCACGTGCGGCCGCACCACGTATACACCATAGCTATC
MuV	1386-N	For: TGCGGCCGCACCTGTGAATCCTTTTGTTCCTCCGGTT
		Rev: ACAGGTGCGGCCGCAACAGGCCCACCTGGTATCC
	1781-N	For: TGCGGCCGCAGGGAGACTGGGATGAGTAAATCACTGACA
		Rev: CTCCCTGCGGCCGCACTCCCACTTGCAACTGTGCGTT
	1858-UTR-N	For: TGCGGCCGCAGCTAACTGCCCAAATCCACTACATTCCA
		Rev: TTAGCTGCGGCCGCATTAGCTGTGGCTGGATTGTCCT
	1881-UTR-N	For: TGCGGCCGCATTCCATTCATATTTAGTCTTTAAGAAA
		Rev: TGGAATGCGGCCGCATGGAATGTAGTGGATTTGGGC
	1976-UTR-P	For: TGCGGCCGCAGCAAGCCATGGATCAATTTATAAAACA
		Rev: CTTGCTGCGGCCGCACTTGCCCGGAAAGAAACACG

	1977-UTR-P	For: TGCGGCCGCACAAGCCATGGATCAATTTATAAAACAGGA
		Rev: GCTTGTGCGGCCGCAGCTTGCCCGGAAAGAAACACG
	1982-P	For: TGCGGCCGCACATGGATCAATTTATAAAACAGGATGAG
		Rev: CCATGTGCGGCCGCACCATGGCTTGCCCGGAA
	2521-P	For: TGCGGCCGCAAAGGAGAGGTCCGGGTCTTTGAGTG
		Rev: TCCTTTGCGGCCGCATCCTTGGACCCAGCTGAGG
	4381-M	For: TGCGGCCGCAATGAGAAACCTATGAGAATTTCCTCTA
		Rev: CTCATTGCGGCCGCACTCATTGAGGCAGACTTCCTG
	4889-F	For: TGCGGCCGCAGGTGTTGCGACCGCAGCACAAGTGA
		Rev: ACACCTGCGGCCGCAACACCGAGCGCAGCAATG
	4905-F	For: TGCGGCCGCACACAAGTGACTGCCGCTGTCTCATT
		Rev: TTGTGTGCGGCCGCATTGTGCTGCGGTCGCAA
	7084-HN	For: TGCGGCCGCAATTTCTCCATCGGCCATCCACTTAACAT
		Rev: GAAATTGCGGCCGCAGAAATCATGGGTTGCATAATCTTC
	12587-L	For: TGCGGCCGCAACCTGTCGAGTCTTGTGTAGTAAATCCGC
		Rev: CAGGTTGCGGCCGCACAGGTCTTATACAGCAAGATGAGC
**NDV**	5367-F	For: TGCGGCCGCACGGGAACCTAAATAATATGCGTGCCACCTAC
		Rev: TCCCGTGCGGCCGCATCCCGACTGAAGGTAGAGTTACC
	5383-F	For: TGCGGCCGCAATGCGTGCCACCTACTTGGAAACCTTATCC
		Rev: CGCATTGCGGCCGCACGCATATTATTTAGGTTCCCGACTGA
	11870-L	For: TGCGGCCGCAACACCAGCAAGAATCCTCCGATGAGGGT
		Rev: GGTGTTGCGGCCGCAGGTGTCATCGGTCAATTCTATATTGC
	11872-L	For: TGCGGCCGCAACCAGCAAGAATCCTCCGATGAGGGTAC
		Rev: CTGGTTGCGGCCGCACTGGTGTCATCGGTCAATTCTATA
	11874-L	For: TGCGGCCGCACAGCAAGAATCCTCCGATGAGGGTACCA
		Rev: TGCTGTGCGGCCGCATGCTGGTGTCATCGGTCAATTCTA
	13215-L	For: TGCGGCCGCAATTACTTAGCCCCGATCAAGTGAGCTC
		Rev: GTAATTGCGGCCGCAGTAATGGTTTCACAGCATCCGACAG
