# Supplementary material for: Genome-wide transposon mutagenesis of paramyxoviruses reveals constraints on genomic plasticity
Source: PLoS Pathog. 2020 Oct 9;16(10):e1008877. doi: 10.1371/journal.ppat.1008877 (PMC7577504; doi:10.1371/journal.ppat.1008877)
Supplement: S7 Table — (PDF) [file ppat.1008877.s007.pdf]

S7 Table. SeV, MuV, and NDV rescue transfection parameters.

	SeV	MuV	NDV
Genome (µg)	4.00	5.00	0.8
pTM1-N (µg)	1.44	0.30	0.4
pTM1-P (µg)	0.77	0.10	0.2
pTM1-L (µg)	0.07	0.20	0.2
pCAGGS-T7opt (µg)	4.00	2.00	0.4
OptiMEM (µL)	200.00	200.00	200.0
Plus reagent (µL)	5.50	7.50	2.0
Lipofectamine LTX (µL)	8.90	18.75	5.0
