# Supplementary material for: Genome-wide transposon mutagenesis of paramyxoviruses reveals constraints on genomic plasticity
Source: PLoS Pathog. 2020 Oct 9;16(10):e1008877. doi: 10.1371/journal.ppat.1008877 (PMC7577504; doi:10.1371/journal.ppat.1008877)
Supplement: S8 Table — (PDF) [file ppat.1008877.s008.pdf]

S8 Table. Primers for one-step reverse-transcription and PCR of SeV, MuV, and NDV.

Pair		Forward	Reverse
SeV	1	ACCAAACAAGAGAAAAAACATGTATGGAAT	GGTCACGAACTCCAGCAG
	2	AAGCGCGATCACATGGTC	TCCCGACCTTTCTCCTGAT
	3	TGGTGCACCTCGGGTTG	CATGGTGGACTGCGATCG
	4	CACTCAGCTCTGTGAGAGTA	GTATAGTTGTCTACAGCACATTCATAAG
	5	AGGGTCCAATACGGCAATCT	GTTATTAAGATGTAAGTGCAATATCAGTG
	6	GAAAGGTTCCTTAGGGAAGC	ACCAGACAAGAGTTTAAGAGATATTTATTC
MuV	1	ACCAAGGGGAGAATGAATATGGGATATTG	ATGTGATCGCGCTTCTCGTTGG
	2	TCCGCCCTGAGCAAAGACC	CGAATTACCACCGGTCAAATTTGCTTGA
	3	CCACTGATGCCTATAGGAGAATCAACAA	GAAAAGGATGGAATTCTCGTGCAACC
	4	CCCACTGCAACCTCACCCAAT	CCAGTCTTGCCTTGGACAGCTTATT
	5	GATCTCAGCATATTCATGAAGGATAAGGC	GATGAGCCTGTGTGCAAGTGTAATG
	6	CTCCAATCAACTTCTCCGTTTATGAAACCA	ACCAAGGGGAGAAAGTAAAATCAATTTTTTCTTAAAAGG
NDV	1	ACCAAACAGAGAATCCGTGAGTTACG	GTTGGATTTCGGACCGCATCATAG
	2	GTCTTGAAACAGACATCCTCCATCC	GTGTCATGATGATCTGGGTGAGTG
	3	TCTGAGATTGCGCTCCGCC	TCCGGAGCACACCAAGTGCTAAAT
	4	GATGCAGAGATCACTCACATTCATATCAGT	CGCCTAAACGAGGCAAGCCAATTA
	5	GCAATCGCACACCCCAACGA	ACCGCAACAGGTGCTTCTCTGAT
	6	CACTGCACCTACATAGTAAATTTAGTTGCTGT	ACCAAACAAAGATTTGGTGAATGACGAGAC
