# Supplementary material for: Genome-wide transposon mutagenesis of paramyxoviruses reveals constraints on genomic plasticity
Source: PLoS Pathog. 2020 Oct 9;16(10):e1008877. doi: 10.1371/journal.ppat.1008877 (PMC7577504; doi:10.1371/journal.ppat.1008877)
Supplement: S2 Fig — (A) Total number of reads at each nucleotide position in the MuV genome, regardless of transposon detection, from the input plasmid. (B) Distribution of insertions in a 100nt sliding window in the input plasmid DNA. To-scale schematic of the MuV genome is included at the bottom. (PDF) [file ppat.1008877.s010.pdf]

S2 Fig. Sequencing and transposon coverage of MuV library.

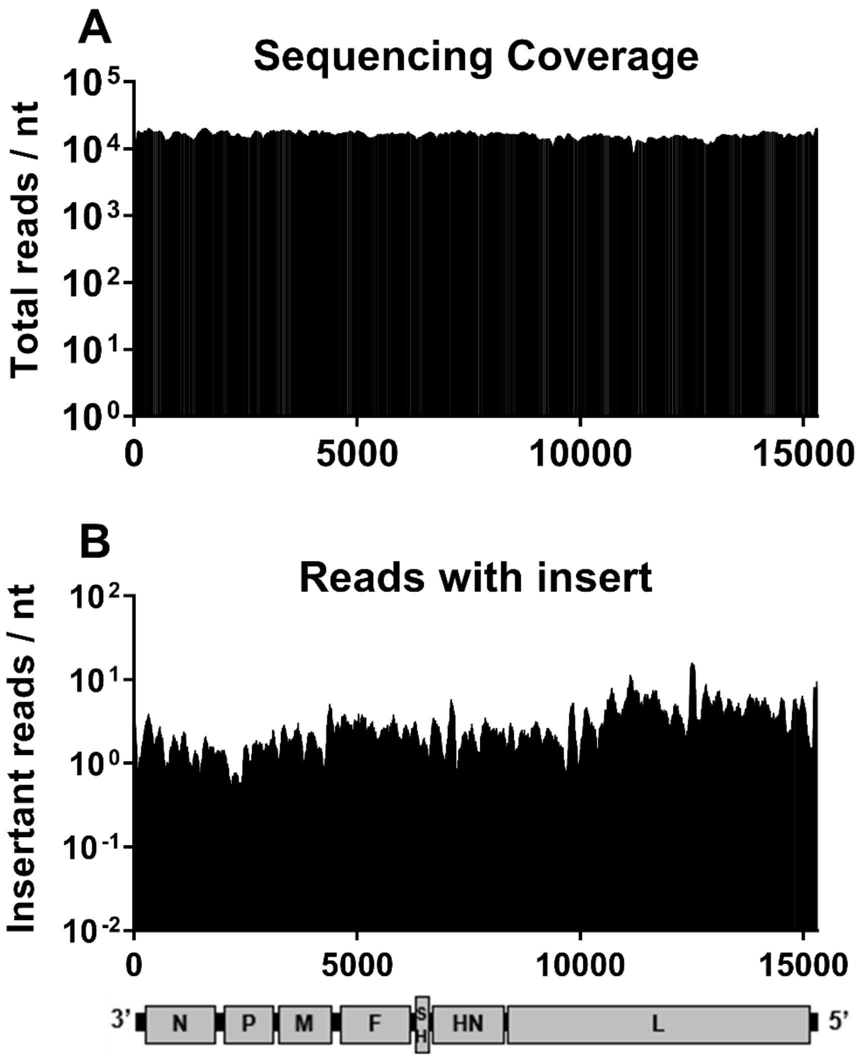

(**A**) Total number of reads at each nucleotide position in the MuV genome, regardless of transposon detection, from the input plasmid. (**B**) Distribution of insertions in a 100nt sliding window in the input plasmid DNA. To-scale schematic of the MuV genome is included at the bottom.
