# Supplementary material for: Genome-wide transposon mutagenesis of paramyxoviruses reveals constraints on genomic plasticity
Source: PLoS Pathog. 2020 Oct 9;16(10):e1008877. doi: 10.1371/journal.ppat.1008877 (PMC7577504; doi:10.1371/journal.ppat.1008877)
Supplement: Attachment — Submitted filename: Ikegame, Beaty et al - SciAdv_Response to Reviewers.docx [file ppat.1008877.s011.docx]

**Authors’ responses to each reviewer comment are in purple text.**

**Reviewer 1**

**Specific Comments**

**1.** *All virus growth studies and passages were done in Vero cells, a simian cell line which is not only very different from natural host cells for SeV, MuV or NDV, but also IFN-deficient. Since the cell type and the lack of an IFN response can have significant implications for the selection of insertants and the so-called fitness hierarchy, a competitive outgrowth analysis in more relevant target cells would definitely strengthen the paper. This would also demonstrate that the experimental approach is not limited to Vero cells and is suitable for testing virus mutants under certain selection conditions in any cell type.*

This is a fair and valid comment. In fact, now that we have shown that it is possible to *rescue* paramyxovirus transposon mutagenesis libraries at the scale (~1 - 3 x10^5 mutants/library) required for informative interpretation; these libraries can be rescued by *any* lab interested in using our method to further interrogate the genomic plasticity of paramyxoviruses under various selection conditions.

We respectfully point out that the rationale for the specific use of BSRT7 and/or Vero cells, which are respectively deficient in interferon signaling and interferon production, was pointed out in the Experimental Design section (lines 443-452) in Materials and Methods. We reasoned that these cell lines "*should serve as neutral cellular backgrounds for our initial genome-wide interrogations. Using these cells removes likely confounding selective pressures against insertions in the P gene that might disrupt its interferon antagonist function (and that of P-derived accessory proteins). We did not want to miss insertants in the P gene that might be structurally tolerated and not affect P’s function as a cofactor of the L-mediated transcriptase and replicase complex*.”

Had we done the initial rescue and passaging of these libraries in IFN-sufficient cells, say A549, the interpretation of the results would be confounded, not only by the reasons stated above, but also by species-specific factors. Since we were interrogating paramyxoviruses that span the three major subfamilies of *Paramyxoviridae* (*Orthoparamyxovirinae, Rubulavirinae, and Avulavirinae*), we chose a cellular background that is arguably the most neutral as most paramyxoviruses have been isolated, rescued, passaged or otherwise attenuated on Vero and BSRT7 cells.

We have now stated this experimental design rationale more explicitly at the beginning of the Results section: “We chose Vero cells – lacking interferon signaling – as a neutral background to remove confounding antiviral selective pressures in our experiments. In particular, the P gene encodes the phosphoprotein, an essential co-factor for the viral transcriptase and replicase, but also encodes accessory proteins to mitigate the host interferon response. In the absence of interferon signaling, insertants that disrupt accessory proteins are not selected against and we can better explore the structural and functional plasticity of the phosphoprotein itself.”

**2.** *The authors conclude that overall, UTRs of the genes tolerated insertions better than the gene ORFs (p6, line 123). Although this conclusion is pleasantly simple and makes sense biologically, I don´t think this can be concluded from Fig. 2E. First, the differences in insertions in the UTRs and ORFs of the M, F and HN genes do not appear to be statistically significant. If they are, the statistics must be shown here. Second and more importantly, the 3´UTR of HN and the 5´UTR of L showed definitively much less insertions compared to their respective ORFs. This must be properly described in the results and must be discussed accordingly (p19, line 19)*

We thank the reviewer for requesting deeper analysis of these statements. We found overall statistical significance for enrichment in UTRs over ORFs in SeV but not MuV, but since this was primarily driven by the insertants surrounding P, we have modified our statements for both SeV and MuV in the results and discussion to focus on regions of the genome (3’ regions) where overall insertant coverage at P2 is sufficient to make confident interpretations.

**3.** *The competitive outgrowth analysis of recombinant insertant viruses shown in Fig. 3 and 5 is a well-chosen assay to test the fitness hierarchy. It allows conclusions about how fit a mutant is when it competes with others. However, since the authors only tested the competitive growth of mutants, the relative fitness compared to wildtype SeV or MuV is unclear. This should be tested in order to determine to what extent mutants can outgrow wildtype viruses, and if this is also tightly correlated with the peak titers.*

This is an interesting question that arose after we completed these experiments. We had deliberately excluded wild-type viruses from the competition assays for two reasons: the first, because we were testing whether the competitive outgrowth assay would reflect the original library pool, which also did not have wild-type viruses; and the second, because we were concerned that wild-type virus would vastly out-compete our insertants, rendering our assay uninformative. Once we completed our analyses, we determined that since among insertants, growth curves and competitive outgrowth assays correlated, we would expect no different were we to include wild-type virus, and so decided it was not a good use of resources in the current environment to re-do these experiments.

**Minor Comments:**

**1.** *The part of the title ´implications for vaccine and gene therapy" should be deleted. The manuscript contains neither data nor a substantial discussion of these aspects.*

We thank the reviewer for noting this, and have removed this section of the title.

**2.** *p4, line 74 What do the authors mean by ´in an otherwise inaccessible fitness landscape´?*

The reviewer is right. Although a fitness landscape is truly what we were exploring, this phrase is opaque without a proper explanation. We have changed the wording to clarify what we mean. Thus, p 4, line 74-75 now reads:

“*Finally, to determine if our experimental strategy had the power to select for mutants and/or insertants that are vanishingly unlikely to occur naturally, that is, in an otherwise inaccessible fitness landscape, . . .* “

**3.** *p8, line 80 The authors might add here ´regardless of whether the PMVs cause systemic*

*or local infections in vivo …´.*

I believe the reviewer here means p4, line 80. Our original intentions were to determine whether receptor usage is a determinant of genetic plasticity. Although the reviewer is correct to note that there is great diversity among paramyxoviruses as to their dissemination in the host, our experiments were carried out *in vitro* and so we chose not to include discussion of the natural history of infection in an organism.

**4.** *p8, line 161 What is meant by ´complex epistatic interactions´?*

Epistasis refers to scenarios in which the effect of a genetic mutation depends on the presence or absence of mutations in other genes; in this instance, we believe that the thousands of insertant genomes in the pooled libraries interact with our chosen insertans, resulting in the seemingly-stochastic differences in insertant count in each library. At line 161, we sought to confirm this finding in Mumps virus.

**5.** *p9, line 188 The title says that ´MuV outgrowth assay reflects library fitness hierarchy´. How*

*does this fit to the statement ´the library screen…was not predictive of relative fitness´ (p10, line 13)*

We thank the reviewer for seeking clarity on this point, and have modified the title here to “Mumps virus competitive outgrowth assay reflects threshold viability of enriched insertants in library screen”. We determined the library signal to be essentially binary, such that insertants enriched above a certain threshold are independently viable for replication upon rescue, while insertants that are not enriched over passage are non-viable. The data discussed in this section does support that information, but the real takeaway was intended to be the connection between growth curve and competitive outgrowth assays, so we have modified the header accordingly.

**Reviewer 2**

**1.** *While the presentation and discussion of the results for the Sendai and mumps experiments are well done, the presentation of the NDV study makes it more challenging to understand the overall significance of this portion. It would be helpful to more carefully delineate the specific F insertions that compensate for the mutation, and include this information in a figure, and then also to re-write the last section to indicate earlier that the L mutation was in the context of an F revertant to the original mutation.*

We thank the reviewer for this helpful feedback; we understand that our NDV findings are complicated, and have revisited their presentation, starting from the section “Input transposon distribution drives selection of L-insertants associated with F^mut^-revertants.” We have also provided context for the NDV insertants – “These insertants correspond to the hinge region between domains III and I in the fusion protein, which has been implicated in fusion regulation (17).” Specific details of the insertants – nucleotide position and amino acid sequence – are provided in Supplementary Table S5.

**2.** It would be helpful to include discussion of what is known structurally about the paramyxovirus glycoproteins versus the influenza glycoproteins - do the structural studies provide clues as to the reason for the observed differences? Are there more regions of flexibility in the influenza glycoproteins, for instance?

This has been expanded upon in the second paragraph of the discussion. We do note that the ortho- and paramyxoviral glycoproteins function in significantly distinct manners, and so detailed structural comparisons require significantly more analysis than are in the scope of this work. Finally, when screening influenza viruses for genetic plasticity, because they are segmented viruses the authors previously carried out insertional mutagenesis a single segment at a time. This enabled the authors to evaluate insertants in a single gene at a time, in comparison to our experiments, where fitness was evaluated in the context of insertants throughout the rest of the genome. This makes it challenging for us to establish the detailed profile of fitness in the paramyxoviral glycoproteins required for a fair comparison of structural details, and so this should be addressed in future mutagenesis efforts.

**3.** *The authors suggest that Figure 3F shows “peak titer was tightly-correlated with performance in the competitive outgrowth assay”, but tightly correlated seems to be too strong a claim for a P value of 0.73.*

We thank the reviewer for their careful evaluation of the manuscript. The value shown in Figure 3F is, in fact, an “r” value indicating correlation between the rankings on the X and Y axes. Since values greater than 0.5 indicate a strong correlation (0 indicates no correlation, 1 indicates perfect correlation), we believe that the use of “tightly-correlated” is appropriate here.
